# Effect of methyl cellulose coating on physicochemical properties, porosity, and surface diameter of pistachio hull

**DOI:** 10.1002/fsn3.227

**Published:** 2015-04-27

**Authors:** Zainab Moslehi, Amir Daraei Garmakhany, Maryam Araghi, Marzie Moslehi

**Affiliations:** 1 Islamic Azad University of DamghanDamghan, Iran; 2 Department of Food Science and Technology, Toyserkan Faculty of Industrial Engineering, Bu-Ali Sina UniversityHamadan, Iran; 3 Islamic Azad University of Amol BranchAmol, Iran

**Keywords:** Methyl cellulose coating, peroxide value, pistachio nuts, scanning electron microscopescanning electron microscope

## Abstract

Pistachio is a nut with high consumption that can be affected by aflatoxin contamination. Regarding influence of this fungus on global trade, broad studies in this area seem to be necessary. In this research, pistachio nuts were coated with methyl cellulose at different concentrations of 0.1%, 0.5%, 1%, and 2% by immersion method. Samples were stored in an incubator (25°C) for 4 months. Imaging was performed by electron microscope using SEM method and chemical changes (moisture, iodine, peroxide, and acidic value) were investigated during storage periods. Results showed that variations in storage time and methyl cellulose concentration had significant effect on moisture content and peroxide value (*P* < 0.05). Also, in case of acidic value, a significant difference was observed between treatments so that pistachio at concentration of 2% showed the highest acidic value. The highest iodine value loss was related to a concentration of 0.1% and the lowest value was observed in the control sample.

## Introduction

Pistachio is an edible seed from the pistachio tree grown broadly in hot–dry regions of the Middle East, Mediterranean, and Americas (Garcia et al. 1992). Nutritional value, palatability, easily digestion, high calorie value, vitamins, and minerals are among the characteristics that make pistachio superior to other nuts. However, factors such as harvest, transportation, and processing enhance the risk of fungal infection in pistachio (Salek Zamani 2001). Favorable conditions in terms of temperature, relative humidity, and water activity result in fungal growth and production of secondary metabolites such as mycotoxin. Aflatoxins are toxic substances from mycotoxin group produced by fungi including *Aspergillus flavus*, *Aspergillus parasiticus*, and *Aspergillus nomius* (Sarhang Pour et al. 2010; Daraei Garmakhany et al., 2010). These toxins cause dangerous diseases such as hepatic cancer in humans (Sarhang Pour et al. 2010). In order to inhibit from aflatoxin production, prevention methods should be in use (Shantha and Decker 1994).

Antimicrobial is a kind of active packaging which can enhance storage life of the food product and protect its microbial health (Seydim and Sarkis 2006). Today, studies on packaging of food products are concentrated on biodegradable films such as prepared films from edible proteins of animal and plant origin (zein, gluten, soy, peanut, albumin, gelatin, collagen, casein, and whey protein). Edible coating is a thin layer of natural substances that covers the food product surface and prevents undesirable changes in flavor, texture, and appearance of the food product (Robertson 2006). Methyl cellulose is a chemical substance which cannot be found in nature. It is obtained by methylation of 30% of hydroxyl groups which is soluble in cold water and gives a clear solution. However, it is insoluble in hot water and changes to saturated solution. Due to its hydrophobic nature, methyl cellulose has a high potential for inhibition of water and oxygen penetration and so increases microbial and chemical resistance compared to other cellulose derivatives (Baker 1982; Shahidi et al. 1999). Also, different concentrations of methyl cellulose lead to formation of different film with different oxygen barrier abilities during coating of pistachio nuts. Pistachio is produced in large quantities and exported from Iran; hence, controlling infection in this product is necessary. However, no attempt has been done on coating pistachio by hydrocolloids. Therefore, the aim of this research was to investigate the effects of different concentrations of methyl cellulose on chemical changes in pistachio nuts during 4 months of storage.

## Materials and Methods

### Preparation of methyl cellulose solution

Abbasali pistachio was obtained from Damghan city. Coating was performed by immersion method. To this end, pistachio kernel was randomly divided into 1 kg packages. In a beaker containing distilled water, methyl cellulose at concentrations of 0.1%, 0.5%, 1%, and 2% was gradually added, and after 30–45 min methyl cellulose was completely dissolved in water. After removing air bubbles, methyl cellulose solution was ready for coating (Turhan and Sahbas 2004).

### Coating process

For coating at each concentration, 1 kg of pistachio kernels was immersed in degasified methyl cellulose solution, stirred for 5 min and coated samples were then stored in an oven at 25°C for 3 days in order to dry. One kilogram of coated dried samples was packed in polyethylene bags and stored in an incubator at 25°C for 4 months (Maftoonazad and Ramaswamy 2005).

### Sample assay

In order to evaluate the effect of methyl cellulose concentration on chemical and quality attributes of pistachio nuts, moisture content, peroxide, acidic, and iodine values were measured. Assays were carried out at the beginning of storage time (immediately after coating and drying), in the middle (after 2 months of storage) and at the end of storage (after 4 months of storage).

### Statistical analysis

This study was conducted in a completely randomized design (CRD) and Tukey's follow-up test was used to determine the difference between treatments (*P* < 0.05). Statistical analysis was done using IBM- SPSS (version 19, Armonk, NY, USA). All experiments were performed in three replications.

## Results and Discussion

### Moisture content

**Figure 1:**
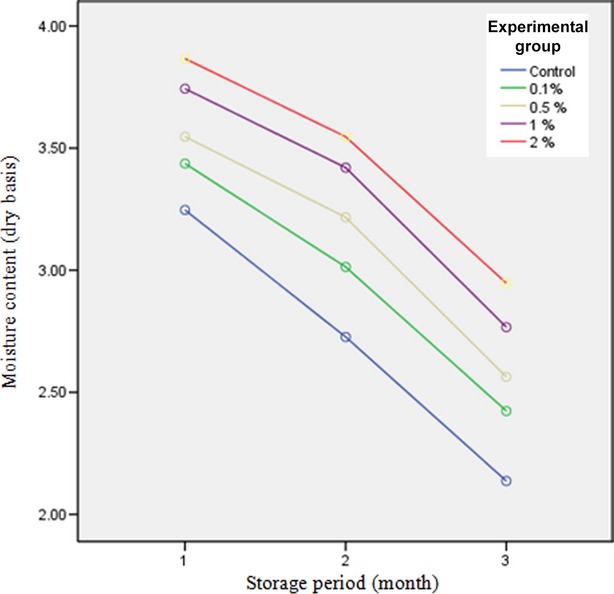
Moisture content of different pistachio-coated samples during storage period.

Based on the results obtained from measurement of pistachio moisture content (Fig.1), the effect of concentration and storage time on moisture content was found to be significant (*P* < 0.05). The highest moisture content was related to pistachio kernel coated with 2% of methyl cellulose solution and the lowest amount was related to control, 0.1% and 0.5% samples, respectively. The reason for this difference is that when pistachio kernels are immersed in methyl cellulose solution, they absorb some parts of the solution through dispersion phenomenon and pistachio kernels coated with 2% of the solution have more density, lose lower moisture content during drying in an oven as well as during the storage period. The moisture content of the food product is decreased with storage time due to moisture exchange with the environment depending on the environment condition (whether free space or inside a package). It is clear that moisture loss in open-packaged food products is lower, depending on the type of packaging material, and other factors such as temperature, exposure to direct sunlight, and nature of the food product. Results showed that moisture loss was decreased due to type of packaging, unique characteristics of methyl cellulose coating, and particular conditions of storage. Several studies showed reduction in moisture loss due to coating and packaging in food materials. Achour et al. (2003), Ur- Rehman (2006), and Babarinde and Fabunmi (2009) reported reduction in moisture loss in packaged dates. Razmkhah (2012) observed that coated pistachio with chitosan had lower moisture loss in comparison with uncoated samples.

### Peroxide value

Peroxide value is the initial product of oxidation of fat substances which does not lead to undesirable flavor and aroma directly but shows the degree of oxidation progress. Peroxide formation proceeds slowly in the initial stages. However, in the secondary stages, it acts as a catalyzer in oil oxidation. More than 89% of pistachio fatty acids are unsaturated. This amount of unsaturated fatty acid leads to enhancement of nutritional value of pistachio but makes it susceptible to autoxidation (Maskan and Karatas 1999). Peroxide measurement was done according to the Shantha and Decker (1994) method. The beginning of this research coincided with the harvest season in order to use fresh pistachio. Moreover, heavy polyethylene packages which were oxygen barriers were used and stored in an incubator in order that storage temperature is constant and pistachio packages are away from direct sunlight. Rate of rancidity and browning reactions resulted from lipid oxidation were reduced in the food products that coated with methyl cellulose (Brenji Ardestani et al. 2008).

**Figure 2:**
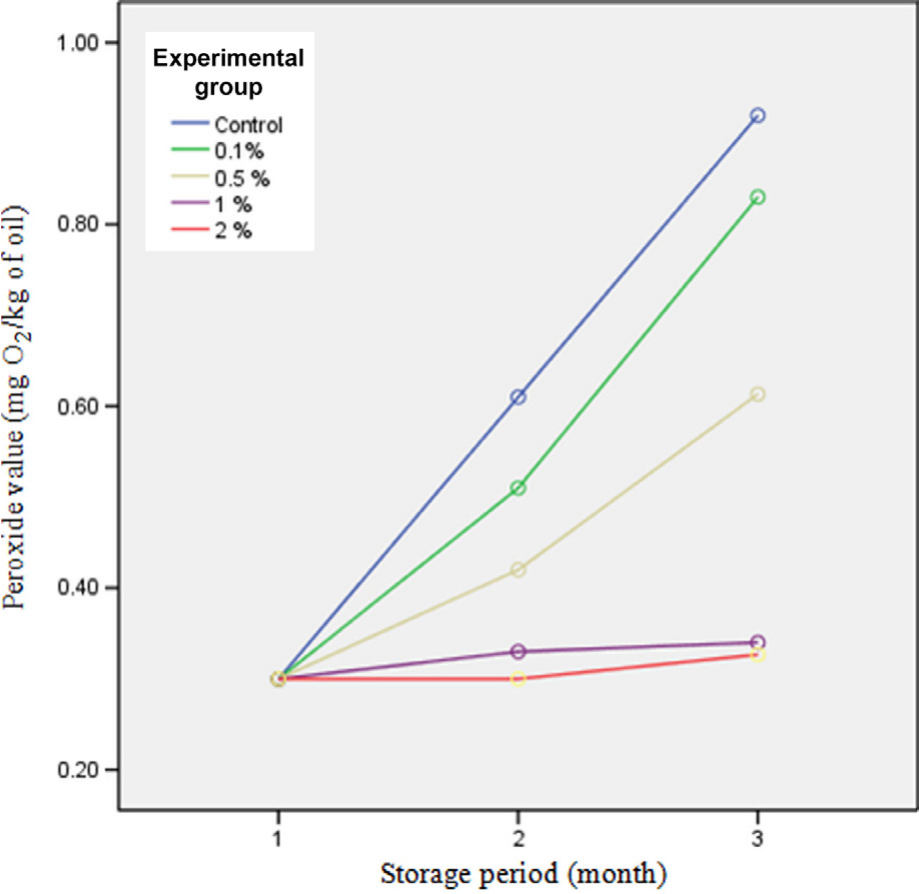
Peroxide value of different pistachio-coated samples during storage period.

As can be seen from Figure2, after 4 months of storage, a significant difference in peroxide value was observed between samples coated with different concentrations of cellulose (*P* < 0.05). The highest peroxide value was related to control and the lowest was related to the sample coated with a concentration of 2% methyl cellulose. It can be explained that in coated nuts methyl cellulose acts as a barrier agent and inhibits oxidation. In addition, by increasing coating concentration barrier efficiency is increased. On the other hand, pistachio oil has a high oxidative stability and peroxide enhancement in this oil proceeds in a lower rate (Mazinani et al. 2011). Yen and Liu (1997) found that substances extracted by ethanol (ethanolic extract) can reduce oxidation of linoleic acid to 80%. This study showed that antioxidant activity of these substances is higher than tocopherol but lower than butylated hydroxyanisole (Yen and Liu 1997). In spite of studies done on the antioxidant property of these products, there is little information about the role of dietary antioxidants. Bressa et al. (1996) separated different compounds from butter-containing cookies which were able to stop autoxidation reactions in the initial stages. In their studies on peanut packaging, Duan et al. (1997) reported a positive role of this process in inhibition of oxidation of peanut oil. Razmkhah (2012) showed an increase in peroxide value in chitosan-coated pistachio samples.

### Acidic value

**Figure 3:**
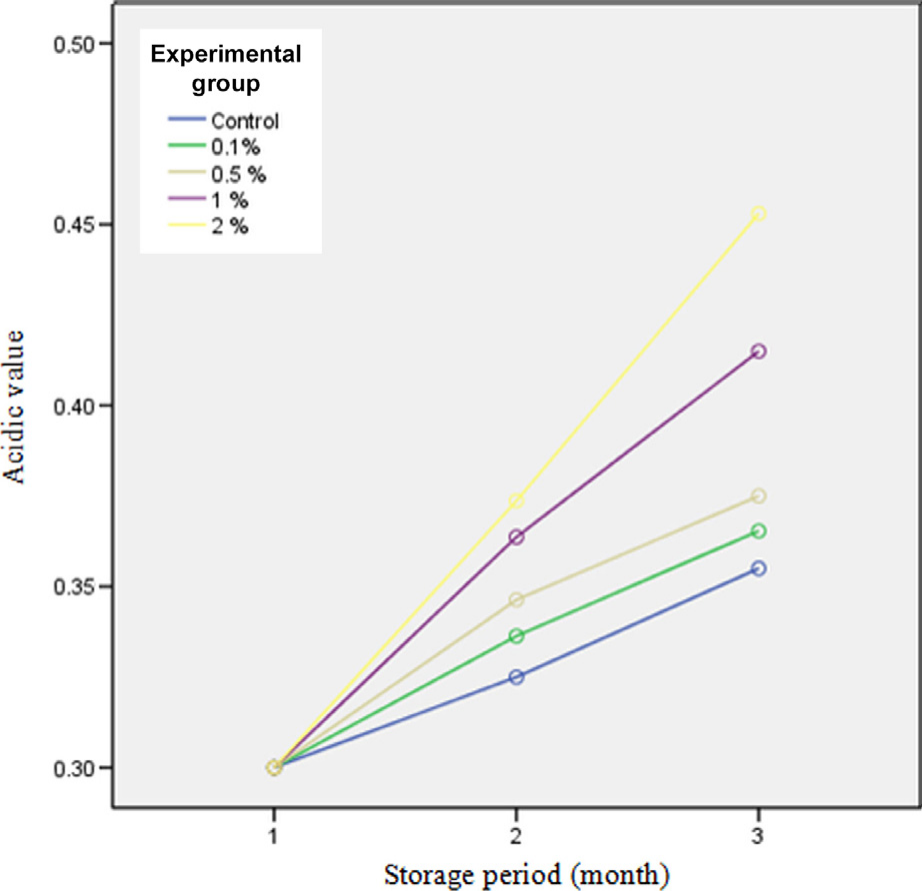
Acidic value for different pistachio-coated samples during storage period.

Acidic value is an indicative index for oil quality (Hill and Hanna 1990). Pistachio acidity was measured according to the AOCS standard (1993). Results showed that there was a significant difference in terms of acidic value between different concentrations of coating solution during storage time (*P* < 0.05). As shown in Figure3, the highest acidic value was related to pistachio samples coated with 2% methyl cellulose and the lowest amount was related to control sample, pistachio samples coated with 0.1% methyl cellulose and pistachio samples coated with 0.5% methyl cellulose, respectively. Increase in the amounts of free fatty acids levels the hydrolysis process in pistachio oil. Lipase and strase cause oxidative reactions of catalase enzyme. These two enzymes separate fatty acids from fat and produce free fatty acids. Therefore, free fatty acids can act as substrates of oxidation reactions. During storage periods the amounts of free fatty acids in pistachio are lower than other nuts; it may be due to the presence of antioxidants in this fruit that inhibit oxidation process propagation (Grosch et al. 1983). At a given temperature, increase in moisture content enhances hydrolysis of fats and production of fatty acids. As described in the previous section, pistachio nuts coated with higher concentrations of methyl cellulose retain a higher amount of moisture content during storage. It can be expected that the lipid oxidation rate in coated samples with a higher concentration of methyl cellulose would be higher than that of uncoated and coated samples with lower concentration of methyl cellulose. It means that moisture has a positive effect on lipolysis and induces this reaction (Reid 1992).

### Iodine value

Iodine index indicates the amount of absorbed iodine for 100 g oil sample. It is regarded as an index for unsaturation degree of unsaturated fatty acids like oleic acid (Mirnezami Ziabari and Saneii 1995; Kirk & Sawyer, 1995). Iodine value was measured according to AOAC standard (1995).

**Figure 4:**
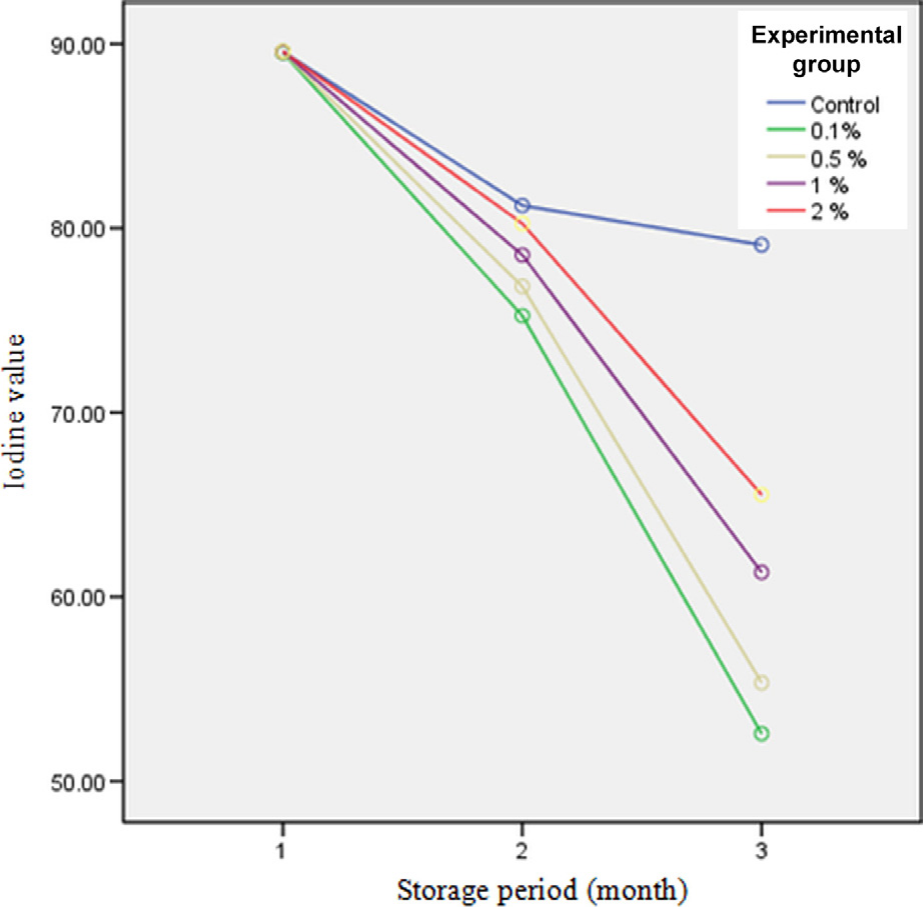
Results of iodine value test for different pistachio-coated samples during storage period.

Results showed that there was a significant difference in iodine value between coated and uncoated samples. As can be seen from Figure4, during 4 months of storage, coated pistachio samples with 0.1% methyl cellulose had the highest loss in iodine value while control sample had the lowest iodine value loss. Moreover, a significant difference was observed between treatments in iodine value during storage time. Iodine value loss during storage periods may be due to reduction in unsaturated fatty acids of oil and consequently destruction of double bands resulting from isomerization, oxidation, and polymerization. As shown in Figure4, a slight increase in the amount of oleic acid led to a decrease in the amounts of linoleic and linolenic acids, thus reducing the iodine value (Qavami et al. 2002).

### Taking image of pistachio nuts under electron microscope by SEM method

#### Coating diameter

**Figure 5:**
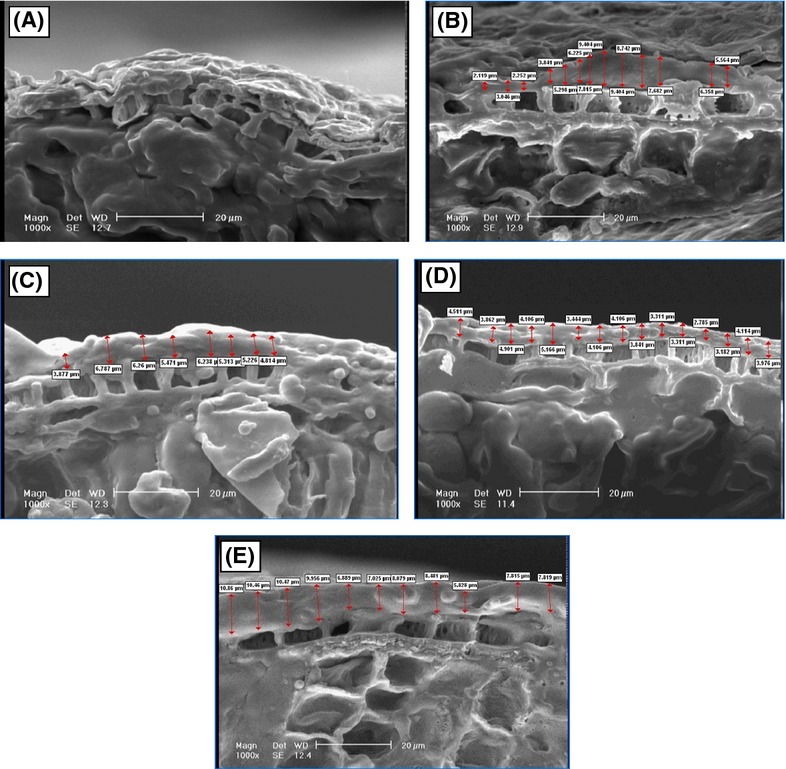
Diameter of pistachio nut samples: (A) control; (B) coated with 0.1% of methyl cellulose; (C) coated with 0.5% of methyl cellulose; (D) coated with 1% of methyl cellulose; (E) coated with 2% of methyl cellulose.

Figure5A–E indicates that all of the coatings have a good density. Based on mean comparison between different samples, coated sample with 1% methyl cellulose has lower diameter than other samples, but it shows the highest density and viscosity. The reason for this low diameter is high viscosity and density of layers at this concentration.

#### Coating uniformity

**Figure 6:**
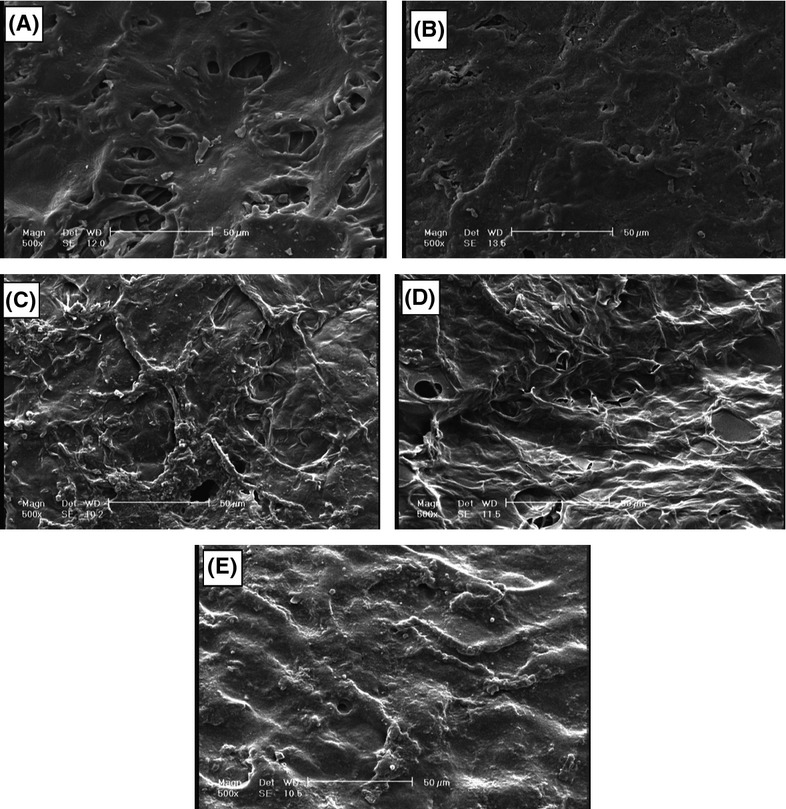
Coating uniformity: (A) control sample; (B) 0.1% sample; (C) 0.5% sample; (D) 1% sample; and (E) 2% sample.

Coating uniformity can be evaluated through investigation of coating surface roughness. It should be mentioned that to obtain electron microscopic images, different parts of the surface and cross sections of samples were investigated. Investigation of cross section surface indicates that sample surfaces are thoroughly coated and there is no fracture. As shown in Figure6, the surface roughness of samples is different. In general, coated samples in D and E are more uniform than those in B and C while sample in E was more uniform than that in D (Fig.6).

#### Size of coating porosities

**Figure 7:**
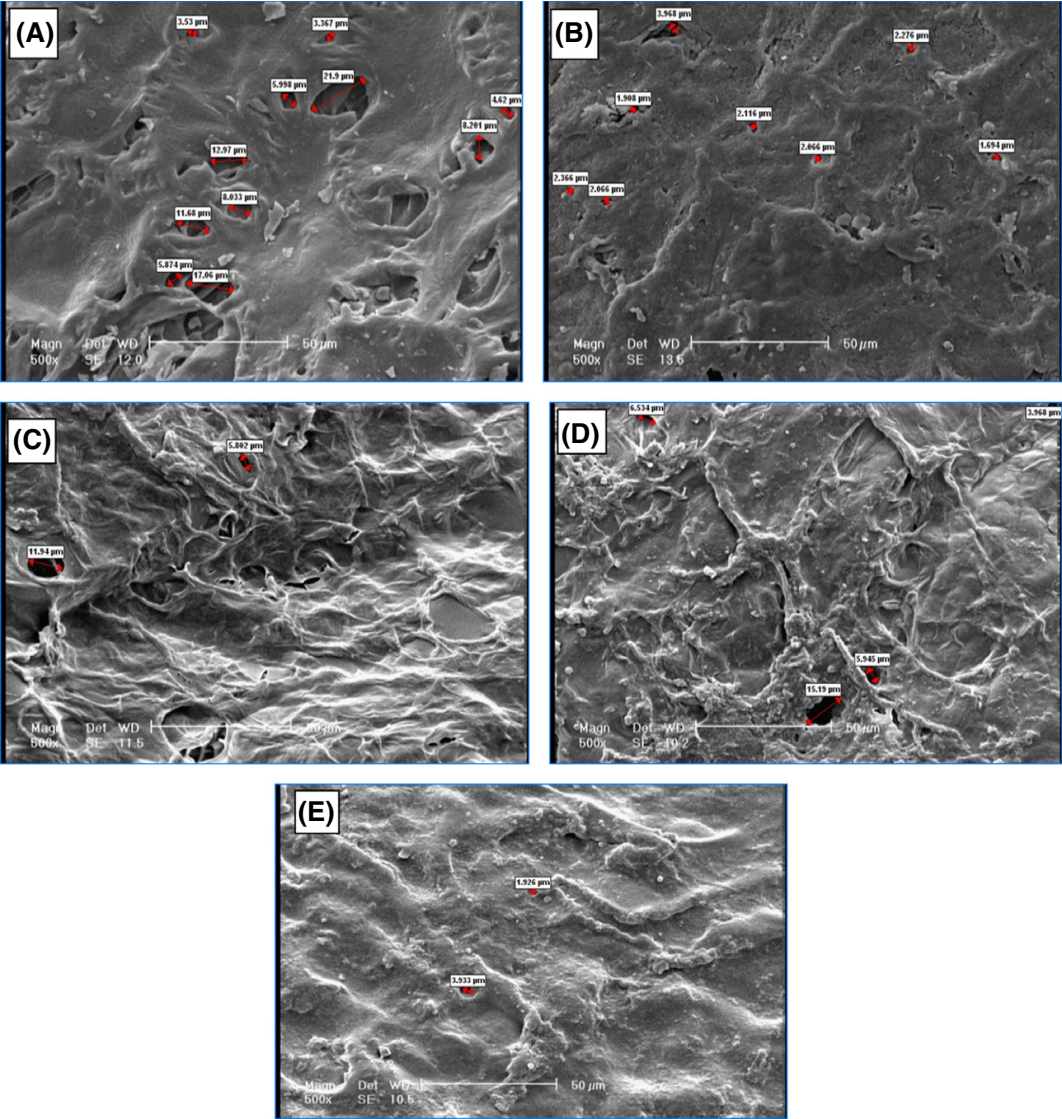
Coating porosity: (A) control sample; (B) 0.1% sample; (C) 0.5% sample; (D) 1% sample; (E) 2% sample.

As porosity determination in imaging software is based on resolution degree, images should be selected in a way that only porosity can be measured. Therefore, inverted electron images (BSE) as well as secondary electron images should be taken from a certain point in order to identify real porosities by comparing them. On the other hand, color change in inverted electron images is due to phase change and presence of porosity. Therefore, to obtain inverted electron images, sample surface should not be uneven. As the cross section surface of pistachio sample is not even (to obtain an even sample, its surface should be polished) and as porosities are well detectable in secondary electron images, images were obtained by a secondary electron detector. Results obtained from images of Figure7 are indicated in Table1. These images were taken by image tool software. As shown in Figure7 and Table1, a coated sample is more porous than other samples and E coated sample has the lowest porosity.

**Table 1:** Size of coating porosity

Sample code	Porosity percent
Control sample	4.3
0.1% coated sample	0.6
0.5% coated sample	1.9
1% coated sample	1
2% coated sample	0.6

## Conclusion

1. With respect to images taken from sample surface, D and E coated samples are more uniform than other coated samples.
2. Investigation of cross section surface of samples indicates that E coated sample has higher density and viscosity than B, C, and D coated samples. (B = 0.1% sample; C = 0.5% sample; and E = 2% sample).

Appropriate coating is a coating with higher density and viscosity, has a surface of minimum roughness and higher uniformity, lower porosities, and smaller mean size of porosity. With respect to these facts and based on chemical tests, images taken by electron microscope and tables related to properties of pistachio samples (surface, cross section surface, diameter, uniformity, and porosity size), it can be concluded that pistachio sample coated with 1% methyl cellulose is the best sample.

## Conflict of Interest

None declared.

## References

[b2] Achour M, Amara S, Salem N, Jebali A, Hamdi M (2003). Effect of vacuum and modified atmosphere packaging on Deglet Nour date storage in Tunisia. Fruits.

[b100] AOAC (2005). Official methods of analysis.

[b101] A.O.C.S (1993). Official methods and recommended practices of the American Oil Chemists Society.

[b3] Babarinde GO, Fabunmi OA (2009). Effect of packaging materials and storage temperature on quality of fresh okra (*Abelmoschus esculentus*) fruit. Agricultra Tropica et Subtropica.

[b4] Baker CA (1982). Methylcellulose and sodium carboxy methyl cellulose: Uses in Paper conservation.

[b5] Brenji Ardestani S, Hossein Azizi M, Zohorian G, Hadyan Z, Amiri Z (2008).

[b6] Bressa F, Tesson N, Dalla Rosa M, Sensidoni A, Tubaro F (1996). Antioxidant effect of maillard reaction products. J. Agric. Food Chem.

[b102] Daraei Garmakhany A, Zighamian H, Sarhangpour R, Rasti M, Aghajani N (2011). Occurrence of Aflatoxin M1 in Raw and pasteurized milk in Esfahan province of Iran. Minerva Biotechnologica.

[b7] Duan S, Zhang JY, Dong XW, Cao GF, Weng XC (1997). Effects of roasting conditions on the quality of fragrant ground nut oils. J. Chin. Cereals Oils Assoc.

[b8] Garcia JM, Agar IT, Streif J (1992). Fat content and fatty acid composition individual seeds of pistachio varieties grown in turkey. Garten bauwissenchaft.

[b9] Grosch W, Laskawy G, Senser F (1983). Storage stability of roasted hazelnuts CCB review for chocolate. Confectionery and Bakery.

[b10] Hill GM, Hanna WW (1990). Nutritive characteristics of pearl millet grain in beef cattle diet. J. Anim. Sci.

[b103] Kirk RS, Sawyer R (1997). Pearson's composition and analysis of foods.

[b12] Maftoonazad N, Ramaswamy HS (2005). Postharvest shelf-life extension of avocados using methyl cellulose-based coating. LWT Food Sci. Technol.

[b13] Maskan M, Karatas S (1999). Storage stability of whole-split pistachio nuts (*Pistachio vera* L.) at various conditions. Food Chem.

[b14] Mazinani S, Elhamirad AH, Piravivanak Z, Naqvi MR (2011). Investigation of thermal resistance, antioxidant properties of phenolic compounds and fatty acid profile in oil extracted from edible nuts (pistachio, walnut and almond). Sci. Mag. Food Sci. Tech.

[b15] Mirnezami Ziabari HV, Saneii M (1995). Common methods of fats and oils decomposition.

[b17] Qavami M, Qarachorlo M, Ezzatpanah H (2002). Effect of frying on qualitative properties of oil used in potato chips industry. Sci. Mag. Agric. Sci.

[b18] Razmkhah M (2012).

[b19] Reid DS, Singh RP, Kusumah MAW (1992). Water relations of foods. A key to product stability. Advances in Food Engineering.

[b20] Robertson GL (2006). Food packaging: principle and practice.

[b21] Salek Zamani M (2001). Nuts 7 their hygienic, nutritional advantages & daily usage. Stand. Monthly Mag.

[b22] Sarhang Pour R, Rasti M, Zighamian H, Daraei Garmkhani A (2010). Occurrence of aflatoxins in pistachio nuts in Esfahan province. J. Food Saf.

[b23] Seydim AC, Sarkis G (2006). Antimicrobial activity of whey protein based edible films incorporated with oregano, rosemary and garlic essential oils. Food Res. Int.

[b24] Shahidi F, Arachechi JKV, Jeon YJ (1999). Food application of chitosan. Trends Food Sci. Technol.

[b25] Shantha NC, Decker EA (1994). Rapid, sensitive, iron-based spectrophotometric methods for determination of peroxide values of food lipids. J. AOAC Int.

[b26] Turhan KN, Sahbas F (2004). Water vapor permeability, tensile properties and solubility of methylcellulose-based edible films. J. Food Eng.

[b27] Ur- Rehman Z (2006). Storage effects on nutritional quality of commonly consumed cereals. Food Chem.

[b28] Yen GC, Liu ML (1997). Antioxidant xylose-lysine maillard reaction products and its fractionated products. J. Chin. Chem. Soc.

